# Supplementary material for: Glucose to Platelet Ratio: A Potential Predictor of Hemorrhagic Transformation in Patients with Acute Ischemic Stroke
Source: Brain Sci. 2022 Aug 31;12(9):1170. doi: 10.3390/brainsci12091170 (PMC9496698; doi:10.3390/brainsci12091170)

Supplement Figure S1. The ROC was used to compare the predictive ability of the G/P, glucose, and platelet in HT. ROC, receiver operating characteristic curve; G/P, glucose to platelet ratio; HT, hemorrhagic transformation; AUC, area under the curve.

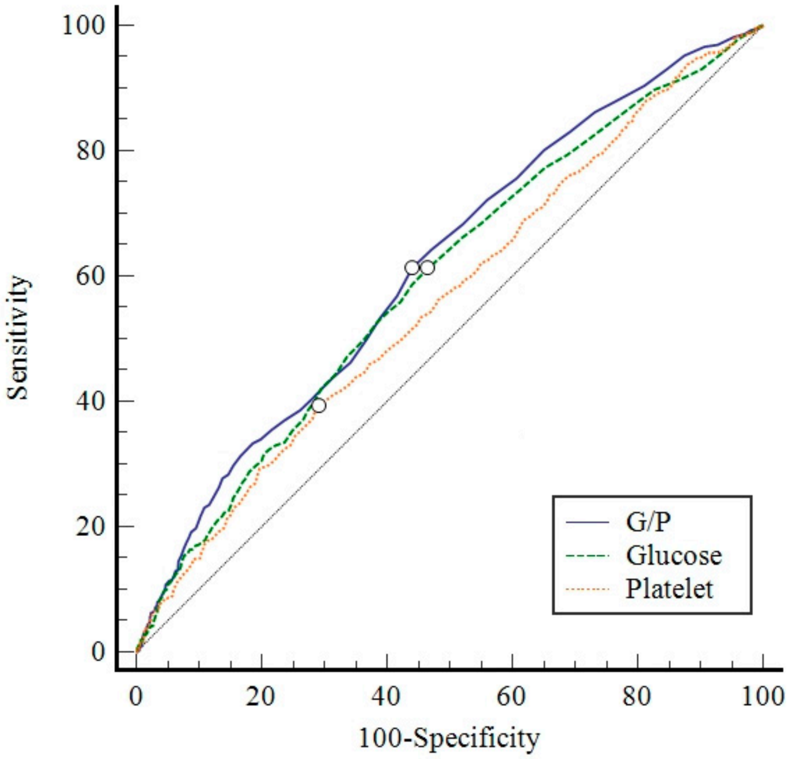

Supplement: Supplementary file 1 [file brainsci-12-01170-s001.zip › brainsci-1860918-supplementary.pdf]
